# Risk of pneumonia associated with zero‐degree head positioning in acute ischemic stroke patients treated with intravenous tissue plasminogen activator

**DOI:** 10.1002/brb3.425

**Published:** 2016-01-19

**Authors:** Paola Palazzo, Amy Brooks, David James, Randy Moore, Andrei V. Alexandrov, Anne W. Alexandrov

**Affiliations:** ^1^ Department of NeuroscienceS. Giovanni Calibita‐Fatebenefratelli HospitalRomeItaly; ^2^ University of Alabama at BirminghamBirminghamAlabama; ^3^ Department of NeurologyUniversity of Tennessee Health Science CenterMemphisTennessee; ^4^ Australian Catholic UniversitySydneyNew South Wales

**Keywords:** Acute ischemic stroke, head of bed positioning, hospital‐acquired pneumonia

## Abstract

**Introduction:**

In the hyperacute phase of ischemic stroke, a 0° position is recommended to increase cerebral perfusion in nonhypoxic patients able to tolerate lying flat. However, use of 0° positioning is not uniformly applied in clinical practice, most likely due to concerns of aspiration pneumonia. We aimed to determine the risk of pneumonia associated with 0° head of bed positioning in acute stroke patients treated with thrombolytic therapy.

**Methods:**

A retrospective descriptive study was conducted using prospectively collected, consecutive acute ischemic stroke patients treated with IVtPA whose head of bed was positioned at 0° for the first 24 h. Rates of hospital‐acquired pneumonia were determined using a strict adjudication process to insure accuracy of pneumonia diagnoses. Quantitative characteristics were analyzed in SPSS to compare differences between “true” pneumonia cases and nonpneumonia cases.

**Results:**

Twenty‐four of 333 (7.2%) patients had mention the diagnosis of pneumonia in the registry and/or medical record. Of these cases, only 15 (4.5%) met evidence‐based diagnostic criteria for hospital‐acquired pneumonia. The 15 adjudicated cases had similar median admission NIHSS scores to nonpneumonia cases (10 vs. 9, respectively; *P* = ns), but were older (74 ± 15 vs. 64 ± 17 years; mean difference 9.889, 95 CI = 1.2–18.6; *P* = 0.026). A total of eight patients with pneumonia were intubated and mechanically ventilated, and one patient received bilevel positive airway pressure ventilation during the 0° positioning period. Pneumonia cases had significantly longer hospitalizations (14.5 ± 12 vs. 6.6 ± 9 days; mean difference 7.97, 95% CI = 1.1–14.8; *P* = 0.026) and higher median discharge mRS score (4 vs. 3: *P* = 0.003).

**Conclusions:**

Zero‐degree head of bed positioning in the first 24 h following an acute ischemic stroke treated with IV‐tPA was associated with acceptable rates of pneumonia. Rates for pneumonia may be further reduced by eliminating use of a 0° protocol in intubated/mechanically ventilated patients.

## Introduction

Early acute stroke therapy is aimed at minimizing neurological deficits with the goal of preventing stroke related disability and mortality (Jauch et al. 2013). Reperfusion thrombolytic therapy with intravenous tissue plasminogen activator (IVtPA) given within 4.5 h of stroke onset (Hacke et al. 2008; Jauch et al. 2013), and more recently, use of intra‐arterial stentrievers following IVtPA administration (Berkhemer et al. 2015; Campbell et al. 2015; Goyal et al. 2015; https://clinicaltrials.gov/ct2/show/NCT01657461) are methods with demonstrated efficacy in reducing disability at 3 months from acute ischemic stroke. Use of a 0° head of bed (HOB) position has been recommended in recent U.S. guidelines as adjunct therapy to further support optimal brain perfusion in acute nonhypoxic, ischemic stroke patients able to tolerate lying flat (Jauch et al. 2013). However, acceptance of this recommendation varies within clinical sites due primarily to concerns for aspiration pneumonia, with some stroke centers favoring use of historical 30° recommendations for HOB position (Schenk 1995).

Several small studies of 0° HOB positioning have produced similar findings tied to improved poststenotic arterial blood flow in patients with large vessel acute ischemic stroke. In a 2002 pilot study, 11 acute ischemic stroke patients, examined within 48 h of symptom onset, showed increased mean flow velocities (MFV) in the affected middle cerebral artery (MCA) on transcranial Doppler (TCD) when the HOB was lowered from a 30° height, to both a 15° and a 0° HOB position (on average, 9.2% and 3.9% increases in MCA MFV, respectively) (Wojner et al. 2002). Similar findings were confirmed in another sample of 20 acute stroke patients, with an average 20% increase in MFV in the affected MCA after HOB modification from a height of 30° to the 0° position, and neurological improvement was observed in three of these patients (Wojner‐Alexander et al. 2005). Similarly, Hunter and colleagues found an increase in MFVs by a median of 26 cm/s (IQR_21.3 to 35.3), with a change in the HOB angle from 30° to 0°, in incompletely recanalized MCAs 24 h from onset of acute ischemic stroke (Hunter et al. 2011). These findings were further confirmed by Durduran et al. (2009) who noted similar gains in cerebral blood flow (CBF) using diffuse optical spectroscopy when the HOB was reduced from 30° to 15°, and then 0°, with the largest gains in CBF noted when patients were placed in −15° Trendelenburg position.

Other investigators have examined changes when the HOB is elevated from a 0° position in acute ischemic stroke. Schwarz and collaborators observed a decrease in MCA MFVs in both affected and contralateral sides, with contextual decreases in cerebral perfusion pressure, intracranial pressure, and mean arterial blood pressure, after HOB was elevated from 0° to 15° and then to 30° in patients with large, nonmalignant hemispheric stroke (Schwarz et al. 2002). Ali and colleagues tested use of 90° upright positioning in five patients that had demonstrated significant clinical improvement despite persisting arterial thrombosis to see if clinical deterioration could be provoked by postural change; these investigators observed dramatic clinical deterioration in three patients who then were successfully treated with reperfusion therapy (Ali et al. 2006). Lastly, a recent meta‐analysis of HOB positioning levels by Olavarria and colleagues found significant improvement of MFV in patients with acute large vessel stroke placed at 0° (Olavarria et al. 2014).

Pneumonia is a common poststroke infection that is a primary cause of morbidity and is the leading attributing factor for mortality at both 30‐days and 1 year poststroke (Heuschmann et al. 2004). Increased length of hospital stays and higher discharge disability rates have been similarly tied to pneumonia (Finlayson et al. 2011). In the United States, stroke patients developing pneumonia incur hospital expenditures averaging $21,000 USD, compared to $6200 USD on average in patients without pneumonia. In fact Katzan et al. (2007) estimated the annual U.S. hospitalization costs of pneumonia as a complication of stroke as exceeding $450,000,000 with 70% requiring continued outpatient care posthospital discharge. Whether 0° HOB positioning increases the risk for aspiration pneumonia in acute ischemic stroke patients has not been previously studied, but concerns tied to this risk likely drive nonuse of this potentially important adjunct procedure. Therefore, we sought to determine the risk of pneumonia associated with a cohort of 0° HOB positioned acute ischemic stroke patients treated with IVtPA.

## Methods

A retrospective descriptive study was conducted using 3 years of IRB compliant, prospectively collected stroke registry data. The sample consisted of acute ischemic stroke patients treated with IVtPA at a comprehensive stroke center who were positioned with the HOB at 0° for the first 24 h, maintained primarily in a side‐lying position, and kept nihil per os (NPO) during the 0° intervention to reduce aspiration risk. Registry data were verified in the medical record for completeness/accuracy; clinical data consisting of breath sounds, pulse oximetry data, temperature, bedside swallow study results, diet/oral intake, pertinent comorbidities, and airway management methods were collected, and radiographic chest imaging, electrocardiogram, echocardiogram, and laboratory data were reviewed to confirm accuracy of any mentioned cardiopulmonary diagnoses in either the medical record or the registry. The timing of 0° head positioning in relation to pulmonary symptom development and a diagnosis of pneumonia were noted. Patients were included if they were at least 18 years of age, diagnosed with an acute ischemic stroke confirmed by clinical exam and MRI or CT brain imaging, with a National Institutes of Health Stroke Scale (NIHSS) score of 3 points or greater, documented treatment with IVtPA within 4.5 h of symptoms onset, and both a medical order for 0° HOB positioning for the first 24 h and nursing documentation of maintenance of 0° positioning. The American Thoracic Society/Infectious Diseases Society of America (ATS/IDSA) guidelines’ definition of pneumonia was used to define the dependent variable (American Thoracic Society; Infectious Diseases Society of America, 2005); specifically cases meeting the pneumonia definition had to meet the following criteria:

- Documented onset of a new or progressive infiltrate on pulmonary imaging along with the presence of at least two of the following –
  o Fever of 38°C/100.4°F;
  o Purulent sputum;
  o Leukocytosis or leukopenia; and/or,
  o Decline in oxygen saturation.

Only cases of hospital‐acquired (nosocomial) pneumonia (HAP) were included in the study, defined as a pneumonia occurring 48 h or more after admission that does not appear to be incubating at the time of admission.

Cases with mention of the term pneumonia at any time from the day of hospital admission to the point of hospital discharge, were presented to an adjudication panel comprised of vascular neurologists, critical care medicine, pulmonology, and emergency medicine to establish consensus on: (1) Whether the diagnosis of pneumonia met the ATS/IDSA definition (American Thoracic Society; Infectious Diseases Society of America, 2005), and if so, (2) whether the timing of any true pneumonia events were either, (a) definitely associated, (b) possibly associated (unsure/could not be ruled out), or, (c) definitely not associated with 0° HOB positioning in the first 24 h of hospital admission. Quantitative patient characteristic data were entered in SPSS and analyzed using descriptive statistics, Student's *t*‐tests, and Mann–Whitney *U* analyses to determine differences between “true” pneumonia and nonpneumonia cases.

## Results

**Figure 1:**
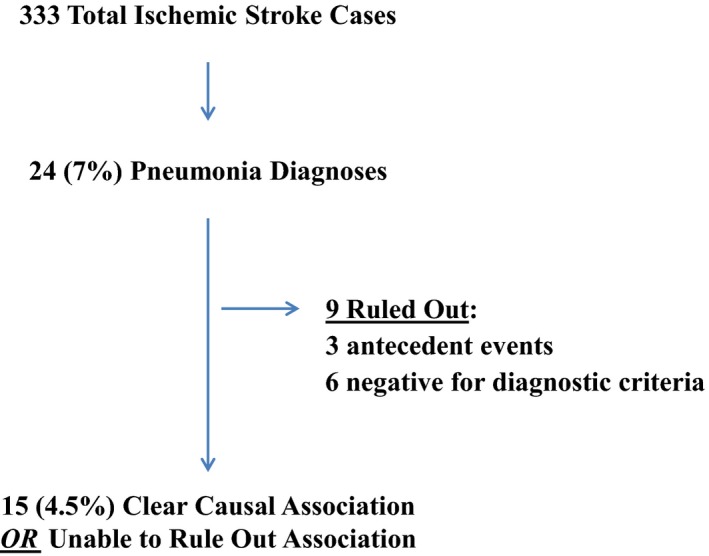
Pneumonia case adjudication.

A total of 333 registry records met inclusion in the study, and of these 24 (7.2%) had mention the term pneumonia in either the medical record or registry (Fig. 1). Adjudication of alleged pneumonia cases identified misdiagnosis of pneumonia in six of the 24 patients when ATS/IDS criteria were applied (American Thoracic Society; Infectious Diseases Society of America, 2005). An additional three patients were removed due to the presence of pneumonia on admission, or documentation of an antecedent event such as vomitus with aspiration occurring in the prehospital environment prior to hospital arrival. Of the remaining 15 (4.5%) cases in the series, 1 (0.3%) case had a clear causal association with 0° positioning represented by vomitus with documentation of aspiration, decreasing pulse oximetry values, and respiratory sound changes during the 24 h 0° HOB positioning period, whereas the other 14 (4.2%) cases could not be ruled out as being associated with 0° positioning in the first 24 h after extensive revision by our team of interdisciplinary experts (Table 1).

**Table 1:** Characteristics of 15 Pneumonia patients

Case	Causal Event for Pneumonia: Confirmed Clinical Event or No Confirmed Event, But No Other Identifiable Cause (Unable to Rule Out)	Age	Median NIHSS	Artificial Ventilation: Intubated/Mechanically Ventilated (I&MV) or Bilevel Positive Airway Pressure (BiPAP) or Independently Breathing (None)
1	Confirmed aspiration event	70	8	BiPAP for sleep apnea associated neurologic worsening
2	Unable to rule out	73	15	I&MV for intra‐arterial rescue
3	Unable to rule out	79	17	I&MV for hypoventilation
4	Unable to rule out	89	13	I&MV for hypoventilation
5	Unable to rule out	36	10	I&MV for intra‐arterial rescue
6	Unable to rule out	93	9	I&MV for hypoventilation
7	Unable to rule out	69	12	I&MV for intra‐arterial rescue
8	Unable to rule out	74	10	I&MV for hypoventilation
9	Unable to rule out	66	8	I&MV for hypoventilation
10	Unable to rule out	84	5	None
11	Unable to rule out	78	9	None
12	Unable to rule out	89	11	None
13	Unable to rule out	75	10	None
14	Unable to rule out	49	9	None
15	Unable to rule out	81	8	None

The remaining 15 pneumonia cases had similar median admission NIHSS scores to nonpneumonia cases (10 vs. 9, respectively; *P* = ns), but were significantly older (74 ± 15 vs. 64 ± 17 years; mean difference 9.889, 95 CI = 1.2–18.6; *P* = 0.026). Additionally, eight pneumonia cases were intubated and received mechanical ventilation during the 24 h 0° period, and one received bilevel positive airway pressure (BiPAP) while positioned at 0°. Pneumonia cases also had longer hospitalizations (14.5 ± 12 vs. 6.6 ± 9 days; mean difference 7.97, 95% CI = 1.1–14.8; *P* = 0.026) and higher median discharge modified Rankin Scores (mRS) (4 vs. 3: *P* = 0.003).

## Discussion

Our study showed that the risk for pneumonia was less than 5% in patients positioned at 0° HOB height in the first 24 h following an acute ischemic stroke. While concern for aspiration is important in the management of acute stroke patients, the actual risk for aspiration pneumonia associated with 0° positioning appears acceptable when patients are kept NPO and primarily positioned side‐lying. In fact, our HAP pneumonia rates are relatively low considering that stroke‐associated pneumonia incidence is 3.9–44% in stroke units (Hannawi et al. 2013) and as many as 30% of stroke patients are reported to be at risk for pneumonia while rates of dysphagia in stroke patients approach 50%, although differentiating true aspiration pneumonias from the general category of nosocomial pneumonia remains controversial (Smith et al. 2015). In addition, in our dataset, 20% of patients with HAP had been intubated to undergo intra‐arterial treatment. The literature suggests that up to 33% of intubated patients fail to be extubated successfully (Kapnadak et al. 2015), and this increases the risk of nosocomial pneumonia. Considering that the new AHA/ASA guidelines propose as reasonable to favor conscious sedation over general anesthesia during endovascular treatment for acute ischemic stroke (Powers et al. 2015), the risk of pneumonia will probably decrease even further as a consequence of reduced intubation rates.

In our study, we deliberately cast a large net to identify pneumonia patients, accepting all cases with a mention of the term pneumonia in either the registry or medical record. Because of this, we believe we minimized the risk of missing actual cases of pneumonia in our dataset. However, these methods also revealed significant inaccuracies in use of the term “pneumonia,” and it is likely that other centers may find similar inappropriate use of this diagnosis. We suggest that cases of alleged pneumonia be cautiously adjudicated using an evidence‐based definition, especially given the implementation of “pay‐for‐performance” quality metrics in the United States that limit reimbursement for hospital services when pneumonia is diagnosed.

Several studies have focused on the development of pneumonia after acute ischemic stroke and have identified specific risk factors. Stroke severity with a NIHSS score ≥10 points has been attributed to pneumonia risk, along with age ≥65 years, dysarthria, aphasia, a mRS ≥4 points, nonlacunar‐basal ganglia infarction, presence of existing infection, intubation, BiPAP use, failed water swallow test (dysphagia), and comorbid conditions which include chronic obstructive pulmonary disease and coronary artery disease (Dziewas et al. 2003; Hilker et al. 2003; Finlayson et al. 2011; Hannawi et al. 2013). Our pneumonia patients had similar risk profiles to those identified in the literature, and the fact that 9 ([60%]; 8 endotracheal intubation/mechanical ventilation and 1 BiPAP) were receiving artificial airway support suggests the need for considering exclusion of these cases in future trials examining clinical improvement in association with 0° positioning.

Zero‐degree HOB positioning may not be tolerated by all patients, specifically those with underlying cardiac and/or pulmonary disease. Modifications of positioning should be considered in these types of patients as well as those at high risk for pulmonary aspiration. Since a 12% increase in MCA MFV has been associated with lowering the HOB from 30° to 15° (Wojner‐Alexander et al. 2005), use of a 15° HOB position might offer a good compromise when positional intolerance is of concern. Use of Trendelenburg has shown the most robust increase in cerebral blood flow in a small pilot study (Durduran et al. 2009), but the utility of this position over time is questionable, and its safety is unknown. Given recent studies demonstrating acute visual loss due to increased intraoptical pressure in patients maintained in negative HOB positions for robotic‐assisted abdominal surgeries (Gkegkes et al. 2015), the risk for further disability in an acute stroke patient would certainly outweigh use of this procedure without judicious ongoing tonometry monitoring.

Additional research is warranted to understand the contribution of 0° positioning as an adjunct intervention to optimize poststenotic arterial blood flow and clinical outcome in acute ischemic stroke. While it is unlikely that use of this position alone is capable of resulting in superior 3 month outcomes, 0° HOB positioning may be an important rescue maneuver until definitive treatment can be provided, similar to procedures used in patients with acute shock states. This could be particularly important in patients with hemodynamic stoke, which have been reported to account for 9.6% of stroke unit admissions (Bladin and Chambers 1994) and are at high risk of neurological deterioration. Patients with significant large artery stenoses, orthostatic hypotension, moderately reduced left ventricular dysfunction, sepsis, and other mechanisms that may challenge optimal perfusion, may be advantaged by this simple maneuver until more definitive treatment can be provided. In fact, in the majority of patients with intracranial arterial stenosis and recurrent neurological deficits induced by positional changes, a significant reduction (≥25%) in intracranial flow velocity distal to the stenosis was observed from resting to symptomatic position, without a significant change in MFV in the unaffected contralateral artery (Saqqur et al. 2010). Lastly, 0° HOB positioning may also improve lytic/clot penetration thereby improving rates of recanalization in patients treated with IVtPA.

Our study has several limitations. First, this was a single‐site study, conducted in a center with a long history of nurses positioning patients at 0° safely. Findings from other sites with less experience in use of this procedure may therefore differ and have higher rates of pneumonia than we found. Also, in all but one HAP patient included in this study (*n* = 14), no data were found in medical records that could tie any clinical events during the 24 h period of zero‐degree positioning to the development of HAP. However, it was felt that these cases could not be excluded due to the retrospective descriptive design of the study. In other words, actual 0° positioning‐related pneumonia rates may be even lower than what we present here, especially when patients with noninvasive ventilation and intubation are excluded. Prospective studies would offer the advantage of immediate case adjudication of true pneumonia diagnoses, confirmation of positional maintenance, and enable collection of serial NIHSS score changes captured over time, offering a better understanding of clinical improvement in relation to 0° position.

In conclusion, while the definitive contribution of 0° HOB positioning to acute ischemic stroke clinical outcomes requires further exploration, our study showed that when used by an experienced team, the procedure is relatively safe. Additionally, we confirm the risk factors identified by others as likely contributors to aspiration pneumonia risk, thereby improving patient selection for this rescue procedure.

## Conflict of Interest

None declared.
